# Flail Chest Resulting from a Rocket-type Firework

**DOI:** 10.5811/cpcem.2018.9.39941

**Published:** 2018-10-17

**Authors:** C. Eric McCoy, Nadia Zuabi, Shahram Lotfipour

**Affiliations:** UC Irvine School of Medicine, Department of Emergency Medicine, Orange, California

## CASE PRESENTATION

**Image 1:**
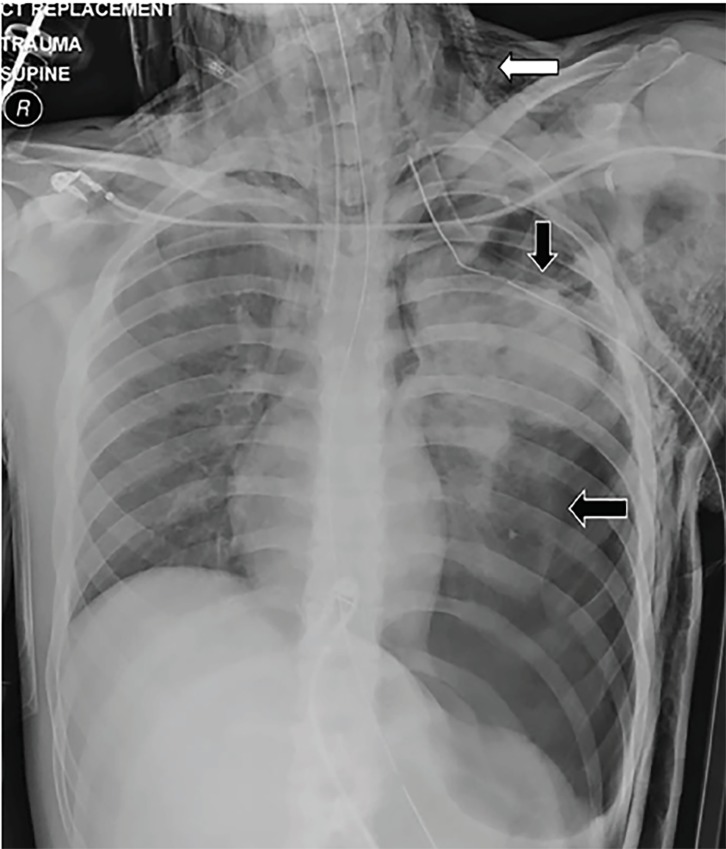
Chest radiograph demonstrating left tension pneumothorax (horizontal black arrow) with near- complete collapse of the left lung as well as significant subcutaneous emphysema (horizontal white arrow) to the left chest wall and neck. Also noted is a chest tube to the left chest (vertical black arrow).

**Image 2:**
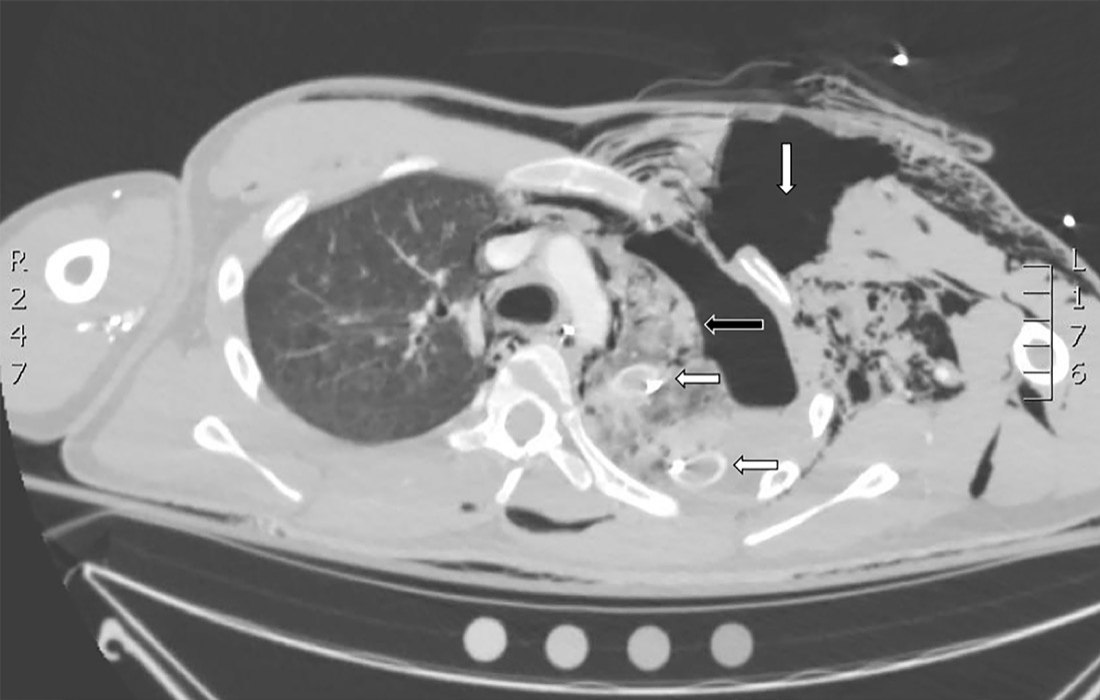
Chest computed tomography scan at the level of the aortic arch demonstrating left-sided pneumothorax (horizontal black arrow) with high-density pleural effusion compatible with hemothorax. There is a large tear of the left pectoral muscles with distraction of the muscle tissue, and a large, air-filled, 5 cm- wide defect (vertical white arrow). Two chest tubes can be seen in the left hemithorax (horizontal white arrow). A large amount of subcutaneous emphysema tracking along the chest wall can also be appreciated.

An 18-year-old male presented to the emergency department (ED) via ambulance after sustaining a blunt force injury to the left chest from a rocket-type firework. He received a needle thoracostomy in the prehospital setting by paramedics after he was noted to be hypotensive with absent breath sounds on the left. Initial ED vitals were temperature of 37.3°C, blood pressure 90 over palpation, heart rate 147 beats per minute, respirations 30 breaths per minute, and oxygen saturation of 89% on 15 liters of oxygen. The left anterior chest wall demonstrated a large ecchymotic burn with powder stippling and an obvious flail segment billowing paradoxically as high as seven centimeters. Subcutaneous crepitation was palpated in the soft tissues of the chest and neck. Tube thoracostomy was rapidly performed and chest imaging obtained (Images 1 and 2).

## DISCUSSION

Chest radiograph revealed a left tension pneumothorax with significant subcutaneous emphysema of the left chest wall and neck. Chest computed tomography revealed additional findings of a large tear of the left pectoral muscles and defects to the underlying intercostal muscles, as well as fractures to ribs 2–4 (Image 2). The patient was taken to the operating room for surgical management.

Flail chest occurs when three or more adjacent ribs are fractured in at least two places, creating a chest wall segment that moves paradoxically from the chest wall.^1^ Flail chest is a life-threatening complication of severe chest trauma with mortality rates of up to 16%.^1^,^2^ Complications may include pneumonia (21%), acute respiratory distress syndrome (14%), and sepsis (7%).^2^ In a review of flail chest injuries in the National Trauma Data Bank, we found that 59% of patients required mechanical ventilation, 82% intensive care unit (ICU) admission, 44% tube thoracostomy, and 21% required a tracheostomy. Although less than 1% of patients require operative management, it has been shown to reduce mortality, duration of mechanical ventilation, ICU and hospital length of stay.^3^–^5^

**CPC-EM Capsule**

What do we already know about this clinical entity?

Flail chest is a life-threatening complication of severe chest trauma with mortality rates of up to 16%.

What is the major impact of the image(s)?

The images demonstrate the significant injuries that can result from rocket-type fireworks sustained at close range.

How might this improve emergency medicine practice?

This case emphasizes the multiple associated injuries and complications to be aware of in patients sustaining close range blunt force chest trauma.

Documented patient informed consent and/or Institutional Review Board approval has been obtained and filed for publication of this case report.
